# How can the integrity of occupational and environmental health research be maintained in the presence of conflicting interests?

**DOI:** 10.1186/s12940-019-0527-x

**Published:** 2019-11-04

**Authors:** Xaver Baur, Colin L. Soskolne, Lisa A. Bero

**Affiliations:** 1 University of Hamburg, Hamburg, Germany; European Society for Environmental and Occupational Medicine, P.O. Box 370514, D-14135 Berlin, Germany; 2 University of Alberta, School of Public Health, 3-300 Edmonton Clinic Health Academy, 11405 - 87 Avenue, Edmonton, AB T6G 1C9 Canada; 3 Health Research Institute, University of Canberra, Canberra, Australia; 4 Medicines Use and Health Outcomes, The University of Sydney, Charles Perkins Centre, D17, The Hub, 6th floor, Sydney, NSW 2006 Australia

**Keywords:** Conflict-of-interest, Public health, Occupational health, Occupational diseases, Research integrity, Ethics, Policy, Public health regulations, Corporate influence

## Abstract

**Background:**

The sciences, and especially the research subspecialties of occupational and environmental health, are being misused. The misuse serves to interfere with the advancement of policies that depend on rational evidence needed for policies to protect public health.

**Methods:**

We selectively surveyed the independent scientific literature. In addition, the efforts of respected international professional organizations of scientists whose focus is on maintaining and improving public health have been considered. This commentary is unique in assembling not only the factual basis for sounding alarms about significant bias in occupational and environmental health research, but also about the manipulative mechanisms used, and, in turn, the methods needed to keep science honest.

**Results:**

Scientific integrity is based on the principle that research is conducted as objectively as possible; it cannot be compromised by special interests whose primary goals are neither to seek truth nor to protect human health. Evidence demonstrates a significant risk of bias in research reports sponsored by financial interests. Practices of corporate malfeasance include the orchestrated contamination of editorial boards of peer-reviewed scientific journals with industry apologists; interference with activities of national regulatory bodies and international review panels engaged in safeguarding occupational and public health; constructing roadblocks by capitalizing on uncertainty to undermine scientific consensus for much-needed government regulation of carcinogenic, endocrine-disrupting and/or immunotoxic agents; promoting “causation” criteria that lack foundation and effectively block workers’ access to legal remedies for harms from occupational exposures resulting in morbidity and premature mortality; and, violating standards of professional conduct by seducing reputable scientists with financial incentives that make them beholden to corporate agendas.

**Conclusions:**

Well-orchestrated assaults on science continue unabated and must now be met head-on. Success could be achieved by promoting and protecting the integrity of research. Furthermore, avoiding influence by conflicted corporate affiliates in occupational and public health regulations is needed. Identifying, managing and, ideally, eliminating corporate influence on science and science policy are needed to protect research integrity. Protecting the public’s health, preventing disease, and promoting well-being must be the unambiguous goals of research in occupational and environmental health.

## Our context

Axiomatic to the question posed in this commentary is that objective knowledge gained through science helps to promote health and longevity. In addition, the freedom of scientists to conduct objective research and share the knowledge gained is essential to the advancement of science in the pursuit of truth.

In practice, the work products of occupational and environmental researchers provide important input to governmental decision-making and regulatory processes on matters pertaining to occupational, environmental and general public health. These researchers have a trusted role to play also in working with the media to inform and educate about scientific knowledge, as well as on the importance of independent science to both public health and safety.

All scientific research contributes to knowledge and must be in accord with the scientific method to be considered valid. Anything that interferes with adherence to the scientific method will serve to erode the integrity of science because anything but valid findings serve to undermine public trust in the role of science for advancing public policy. The integrity of the scientific method is best protected by exposing and blocking the undermining role of special interests that are incongruent with the public interest.

Without access to valid scientific evidence, those in the regulatory domain will not be able to make rational, informed decisions; each of health, safety, social justice, and environment are thereby placed in jeopardy. Derailing science in its pursuit of objective knowledge ultimately demeans science. In turn, the public-policy process and our democratic institutions are negatively impacted, as are the public’s health and longevity. Through continuing education in research methods, ethics, and best practices, science is further advanced. In addition, education serves to hone the skills of scientists for detecting invalid science.

With conflicting roles and opinions between health scientists (e.g., physicians, practitioners, occupational and public health and biomedical researchers) and corporate interest groups (e.g., pharmaceutical, medical device, biotechnology, other industries and insurance companies) being widespread, media outlets are inclined to present opposing viewpoints. In this commentary, we show that a major source of conflict arises because the goals of for-profit companies include producing products that maximize financial returns to shareholders, while the goals of science include the pursuit of truth and the advancement of knowledge, in addition to maintaining and improving public health.

The goal of this commentary is to help expose and rout out invalid science by sensitizing scientists to those business influences that continue to undermine the pursuit of truth through the generation of invalid science.

## The scope of research integrity and conflict-of-interest (COI)

Integrity in occupational and environmental health research, as in other scientific research, requires that the scientist adhere to the goal of pursuing truth. To achieve this, the scientist has a duty to be impartial throughout the process of addressing a research question. The research must not be compromised by special interests.

Providing unbiased knowledge to guide the protection of public health, and thus prevent disease and promote well-being, must be the unambiguous goal of all research and other activities in occupational and environmental medicine and public health. Any scientist who succumbs to influence that detracts from the principle of the pursuit of truth is, by definition, unethical by virtue of producing invalid scientific assessments that fail to serve the public interest, protect public health, or advance science and knowledge. This does not mean that funding from interested parties by itself undermines integrity; however, there is a high risk for bias when research is not well-designed, not properly analyzed, and not objectively interpreted as demonstrated to be the case when research is sponsored by business with financial interests. Consequently, influence has to be recognized and managed with a view to minimizing its impact; ideally, it should be eliminated.

Because of the public interest dimensions of the scientific enterprise, funded in part by the public purse, the United States Public Health Service in 1980 addressed the challenge of misconduct in science by establishing the Office of Research/Scientific Integrity. In their 2017 update, they define misconduct in science as “fabrication, falsification, or plagiarism (FFP) in proposing, performing, or in reporting research” [1]. See the corresponding definition and policy implementation by all US federal government agencies supporting intramural or extramural research through the Office of Science and Technology Policy 2000 [2]. A further category mentioned in the 1980 definition is that of “detrimental research practices”, i.e. actions other than FFP. Actions such as selective publication and inappropriate analysis violate the traditional values of the research enterprise and are detrimental to the research process. Another dimension of misconduct is unacceptable behavior that is not unique to the research environment [3].

Malfeasance can arise when those engaged in science and regulatory processes are put into, or find themselves in a conflict-of-interest (COI) situation. The scientist who fails to acknowledge a COI and who knowingly proceeds to misuse and manipulate the scientific method to produce findings that support the interests of his/her sponsor, would be accused of malfeasance.

The risk of malfeasance arises when a secondary interest (such as that of personal financial gain) could adversely affect a primary interest (such as the duty to produce valid research). A COI may be financial (e.g. stock ownership, consulting fees) or non-financial (e.g. personal relationships) [4]. COI is not in itself a bias or a corrupt decision but, rather, a set of circumstances that poses a risk for primary obligations being compromised by succumbing ─ consciously or even subconsciously ─ to the influence of other interests. The existence of a COI does not imply that a scientist is improperly motivated; his/her perspective, however, may become biased.

Conflicts are not binary; that is, they are not simply either present or absent. A COI can be more or less severe and the seriousness of a conflict depends on the likelihood that scientists and physicians would be unduly influenced by a secondary interest and the degree of the harm or wrong that could result from such influence. See also Table 1 (below).

**Table 1:** Criteria for Assessing the Severity of Conflict-of-Interest (from [55])

Likelihood of undue influence	
• What is the value of the secondary interest?	
• What is the scope of the relationship?	
• What is the extent of discretion?	
Seriousness of possible harm	
• What is the value of the primary interest?	
• What is the scope of the consequences?	
• What is the extent of accountability?	

To minimize the impacts of COI on public health and to provide guidance for formulating and applying COI policies, a framework for analyzing conflicting interests is desirable.

## Our current reality

**Fig. 1:**
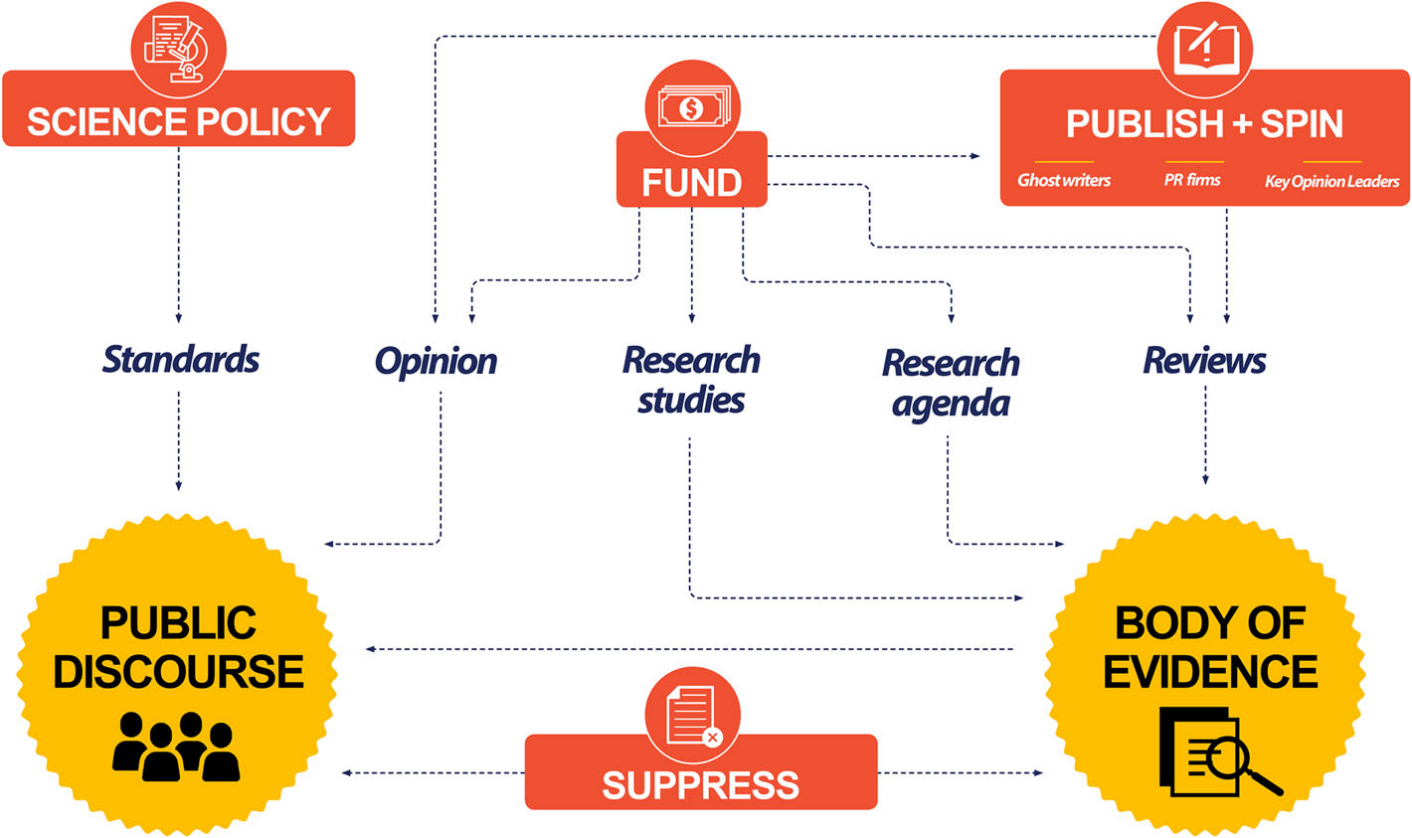
Industry strategies to influence evidence and discourse about evidence ( [8] by courtesy of the author)

The influence of industry on occupational, environmental, and public health research and practice has been well documented [5, 6]. Corporate interests have frequently influenced science by an array of approaches [7]. Specifically, corporate interests influence research agendas and the design, conduct and dissemination of research through a variety of strategies (see Fig. 1).

## Influencing science policy

One common method for influencing science policy arises when academic institutions are influenced through their pernicious pursuit of money and merge their interests with that of industry. Another most disturbing way that industry influences science is by its attempts to change the evaluation of science, particularly for its use in policy [9, 10]. For example, the tobacco industry worked with established and existing business coalitions including the American Petroleum Institute, National Rifle Association, and the American Iron and Steel Institute to legislate changes in how research should be evaluated before it could be cited as evidence in support of a policy [11]. The American Chemistry Council works with politicians and regulators to develop policies that limit what science can be used to evaluate environmental pollutants. Also, through a careful read of the process followed, corporate interests shaped the Brussels Declaration on Ethics and Principles for Science and Society Policy-Making [12] to enhance the ability of industry to influence evidence and policy related to harm reduction. Thus, it surprises us that the Brussels Declaration received favourable support from key scientific organizations, including its launch at the American Association for the Advancement of Science and a letter in Nature.

Another important dimension of science policy malfeasance has arisen with the infiltration by vested interests of governmental decision-making bodies and regulatory processes for assessing hazards, risks and the need for preventive actions. Examples of such conduct include tobacco, asbestos, pollution, climate change and many other issues of commercial interest.

All these activities have been part of the industries’ campaign to promote their version of ‘sound science’ and ‘good epidemiology’ [13–18]. In so doing, corporate interests frequently have inhibited or even blocked legislative solutions to ensure that public-policy is based on sound science [11, 17].

A well-studied example of corporate manipulation and malfeasance relates to the asbestos industry, along with their insurance companies, which for almost a century have influenced the results of scientific findings, delayed important knowledge about the asbestos-cancer relationship, and thereby influenced law and public policy to serve their own interests rather than the interests of workers and the public’s health. Scientifically credible consultants were engaged to cast doubt on adverse health effects, diagnostic criteria and compensation issues; nowadays, the asbestos industry continues this influence [15, 19–24] by promoting the safe use of chrysotile asbestos which is not possible. Their strategy has successfully prevented its banning in various countries and its listing in the Rotterdam Convention declaration [25].

## Influencing research and scientific publication

It is well known that COI has a strong influence on the outcome of research studies, thus shaping science to serve business interests [14, 26–29]. As reported by Friedman and Friedman [46], as well as by others [18, 30–32, 52–55], in cases of financial COI, the proportion of findings supporting business interests differed significantly from studies without COI. Because of hidden ties, including ghost writing that cannot be identified, the real discrepancy can be assumed to be even greater. The chemical, pharmaceutical, car, nutrition and other industries have conducted research to [14, 33] deny, ignore or marginalize the adverse health effects caused by endocrine disrupting chemicals [30–32, 34], glyphosate/Round-up [35], various drugs [14, 33], sugar [36], and the recent dieselgate issue by broadly applied default devices [37, 38].

A further approach is the systematic infiltration of the scientific literature and the media with biased science. This includes creating industry-driven scientific journals which can steer the perception of ‘the evidence’ by favoring studies that underplay or deny risk, giving supposed scientific credibility to editorials or biased reviews that can be used in litigation to defend industry and allow publication practices that bypass acceptance norms for scientific integrity. Detailed information on this aspect is available from the Toxic Docs website of the Columbia University and City University of New York [39] and includes the influence of publishers/journals, new forms of detrimental research practices such as the dismissal of journal editors engaged as health advocates for victims (example/ref. Wiley - D. Egilman [17, 40];), fake peer reviewing by some journal editors, and applying fake and predatory journalism with little or no editorial review or quality control of papers [41, 42]. Furthermore, groups wishing to bias research perception are using information technologies to manipulate how science is understood by consumers. See as an example tweet by President Donald Trump on 12:22–23. Aug. 2012 on “Massive combined inoculations to small children is the cause for big increase in autism...”. https://twitter.com/realdonaldtrump/status/238717783007977473

Obvious examples can be seen in:

- the manipulation of information technology in analysing research data to obtain desired results;
- globalizing a specific view of research findings through the use of “independent experts” commenting via social media, see for example the climate change and global warming issue as repeatedly presented by Donald Trump [43], and
- increasing the irrelevance of knowledge generated in certain fields to policy issues and political debates through a pervasive media environment that can help to generate unsound findings and controversies**.**

Another common tactic is to develop and disseminate public statements claiming that well-established facts are controversial. Several strategies have been adopted by entities with an economic interest in the outcome of health assessments to construct confusion by creating artificial controversy, for instance:

- Establishing principles of so-called ‘good epidemiological practice’ intentionally to be misused to dismiss studies that provide reliable evidence of harm as irrelevant for decision-making processes;
- Promoting impossibly difficult criteria for establishing causal relationships;
- Designing research which does not fit with the principles of sound science, resulting in manipulated research results; and
- Ignoring well-established knowledge on adverse health and/or environmental effects with highly selective interpretations of the literature [19, 31, 44].

## Influencing research agendas

The increase in funding by vested interests reflects the ever-growing domination of the financial world in setting priorities for research. This can be a direct challenge to protecting human health. The relative lack of independent funding and/or lack of access to data poses grave dangers for the future of impartial research conducted in the public interest. This imbalance in funding between private and public sources creates a risk that many scientists in their search for funding make opportunistic or naïve compromises with industries that have an interest in particular research outcomes that support their products or activities [29, 33, 35, 45, 46]. It is not surprising that corporate interests should want to fund research to support their financial interests.

The glyphosate issue is a case example where science is misinterpreted [see Table 2 below].

**Table 2:** Case examples of industry efforts to influence glyphosate regulations [48]

Applied measures to misinterprete science	
• Ghost-written research papers that assert glyphosate safety [35]	
• Provided alternative interpretations of positive studies	
• Used least statistically powerful tests on submitted research	
• Developed a network of scientists to push glyphosate safety and attack IARC	
• Used public relations teams and others to increase political activity and to attack the messenger (IARC, scientists, journalists, etc.)	
• Provided EPA “talking points” about IARC classification	
• Challenged two members of EPA’s SAP and one was removed	
• Enlisted EPA to block ATSDR review of glyphosate that they were worried would agree with IARC.	
• Drafted original Renewal Assessment Report for EFSA	
• Exploited close relationships with journals, journalists and some regulatory staff	

## Further examples of industry strategies

The strategies are broad. Some additional examples follow:

- offering scientists generous resources for research, but with restrictions on publication rights. This serves to absorb research capacity and control the results;
- paying scientists for consultancy and for representing industrial interests in science and policy fora often without disclosing their ties to industry;
- sponsoring pseudo-scientific think tanks, or special issues of journals that present the findings of a series of manipulated studies; and
- conducting *ad hominem* attacks on scientists who have published findings suggesting hazardous associations with industry products or processes.

The intent of the aforementioned corporate activities ─ in decision-making processes for assessing hazards, risks and the need for preventive actions regarding among others, tobacco, asbestos, benzene, diesel exhaust, plastics, pesticides, climate change ─ is to promote self-interest regardless of the cost to the public’s health. One of the best example is the tobacco industry which has backed a number of ‘astroturf’ initiatives to attempt to influence regulation [48]. Far too often, early warnings of occupational and environmental hazards are intentionally delayed or dismissed through being able to maintain the *status quo* and protect business interests.

## Why and how to assess and evaluate COI

Complete and accurate disclosure of financial ties with corporate interests, which is still frequently lacking, is a critical first step to routing out influence in the advancement of knowledge. This would make more transparent the forces of both bias and influence in science.

When conflicts of interest are made transparent, they should be assessed by considering various factors that determine their likelihood and seriousness. Likelihood depends on the value of the secondary interest, the scope of the relationship between the professionals and the commercial interests, and the extent of discretion that the professionals have (Table 1). Seriousness depends on the value of the primary interest, the scope of the consequences that affect it, and the extent of accountability of the professionals. COI policies should be evaluated by considering their effectiveness, transparency, accountability, and fairness in order to deal with such conflicts appropriately [55] (Table 3).

**Table 3:** Criteria for evaluating conflict-of-interest policies (from [55])

Criterion	Description
Proportionality	Is the policy most efficiently directed at the most important conflicts?
Transparency	Is the policy comprehensible and accessible to the individuals and institutions that may be affected by the policy?
Accountability	Does the policy indicate who is responsible for enforcing and revising it?
Fairness	Does the policy apply equally to all relevant groups within an institution and in different institutions?

A better understanding of the nature of COI and a clearer and fairer formulation of rules could support greater confidence in medical and scientific advice and thereby enable researchers to concentrate on their primary missions of conducting and publishing unbiased research.

## How to address the continued undermining of integrity in science and COI

Sound science using policy-making processes and regulations has the potential to solve occupational and environmental health problems, and to impact society in a more sustainable way. Physicians, practitioners, public health and biochemical researchers, advocate based on their credibility. They thus have the duty to speak out on this issue with students, colleagues, peers, in the professional arena (including the scientific literature) and, through the media, to the public.

A major problem is that scientists are frequently not aware of underlying processes and/or are not willing to take part in the time-consuming and cumbersome work of addressing scientific integrity. As stressed by Agerstrand et al. [56], actions for increased understanding about science-policy interactions are urgently needed. These include the reporting of studies in a way that enables their regulatory use, submitting studies and comments on current sociopolitical assessment and processes, dialogues with stakeholders and policy–makers, as well as training the next generation of scientists in public health. A systemic approach addressing perverse incentive structures within universities and editorial boards and a shift towards a rights-based paradigm with genuine stakeholder involvement are recommended [57].

## Principles for safeguarding the integrity of research in occupational and environmental health

The following steps have been proposed to decrease and expose the influence of financial conflicts of interest on the integrity of research in occupational and environmental health and to help inform policy-makers [58]:

### Conflict-of-interest declarations

COI declaration should focus on declarations of financial resources for the research activity, and on any relevant connection of the researchers with industry that might have a financial interest in the outcomes of the study. Effective enforceable disclosure policies including penalties for not disclosing accurately must play an important role in protecting peer review journals, peer review panels, and government entities against becoming unwitting agents of misinformation. However, effective COI disclosure policies are necessary, but are not in themselves sufficient.

### Scientists’ ethical constraints

Ethical constraints of research and the evaluation of the respect of these ethical principles should equally apply to all research activities in the field of occupational and environmental health, no matter who initiates, conducts or finances it. Failure to enforce ethical principles is not acceptable.

### Funding

It is the responsibility of governments to foster the conduct of impartial research of which the primary goal is to discover and communicate relevant evidence on factors affecting workers and population health. Failure to promote such efforts will adversely affect decision-making policies and practices in occupational and environmental health. The creation of independent research funds to which industry must contribute may be a partial solution to this problem.

### Decision-making processes

It is not possible to eliminate the production of all bad science. But it is possible to prevent the use of the outcomes of bad science in decision-making processes and in assessments of health hazards and risks. Fairly evaluating published research in the process of peer-review is becoming increasingly challenging in a world that is characterized by infiltration of powerful interests at all levels of science [60]. Applying the principle of COI declaration for every person involved at each level of decision-making may create the necessary transparency to identify and address distortions by the regulated community. Research evidence that is used to inform policy should be evaluated according to criteria that are the consensus of the independent scientific community, and not the industry being evaluated.

### Restoring dignity in academic publishing

The rise of predatory publishing without quality standards and with commercial interests represents a severe threat to the scientific community and to those who rely on the assumed validity of scientific findings. To preserve the integrity and dignity of being a scientist, every researcher, clinician, academic and professional should scrutinize the journals cited and to which publications are submitted [61].

The Collegium Ramazzini [58], an independent, international academy with internationally renowned experts in the fields of occupational and environmental health, called for the following detailed actions:

- National and international official bodies to set up evaluation procedures that systematically orient funding towards research centers, researchers and research activities with demonstrated commitment to competence and impartiality in assessing health effects.
- Governments to operationalize the Right to Enjoy the Benefits of Scientific Progress, as contained in the International Covenant on Social, Economic and Cultural Rights by promoting science of the highest ethical standard as a public good. That right implies an obligation on government entities to create a research environment in which unbiased and relevant scientific knowledge is advanced and disseminated without obstacle. Efforts to reinterpret science or assessments of it in a biased way that apparently favours economically and politically vested interests could be interpreted as an interference with that right. Public policy-makers and the public can benefit from science only if it is allowed to be conducted, assessed, and communicated in an unbiased way. States should also ensure transparency in funding of research through mandating open declaration of sources of funding when research is proposed, disseminated, and presented.
- Scientific journals to establish mechanisms, consistent with international best practices that provide disciplinary action for editors, authors and peer reviewers who fail to disclose financial conflicts and competing interests [28]. In the absence of effective implementation, policies mean little.
- All public institutions that play a role in risk assessment and public health policies to systematically rely upon the advice that is transparent, credible and subject to public scrutiny.
- All decision-making bodies to set up effective COI disclosure policies for all persons involved in the process.
- The scientists involved in occupational and environmental health to never divert from the path of scientific integrity in their scientific research, assessments and communications, and that they consistently strive for objectivity, impartially pursue scientific truth and eliminate financial and other COIs, with a view to public health protection.
- The Collegium Ramazzini encourages scientists to contact the Collegium when their independence is threatened in a way that puts a burden on their freedom to consistently follow that path.
- The Collegium Ramazzini calls on all professional bodies to support scientists who are under threat for speaking the truth.

Similarly, according to A Consensus Study Report of the National Academies of Sciences, Engineering, Medicine [1] the following activities are needed:

- all professionals in public health should significantly improve and update their practices and policies to respond to the threats to research integrity;
- research institutions should maintain the highest standards for research conduct;
- research institutions and federal agencies should work to ensure that whistle-blowers are protected and that their concerns are assessed and addressed in a fair, thorough, and timely manner;
- a research integrity advisory board should be established as an independent not-for-profit organization;
- societies and journals should develop clear disciplinary authorship standards;
- research sponsors and science, engineering, technology and publishers should ensure that information sufficient for a person knowledgeable about the field and the techniques to reproduce reported results is made available;
- federal funding agencies and other research sponsors should allocate sufficient funds to enable the long-term storage, archiving and access of datasets necessary for the replication of published findings;
- researchers should routinely disclose all statistical tests carried out, including negative findings;
- governmental agencies and private foundations should fund research to quantify, and develop responses to, conditions in the research environment that may be linked to research misconduct and detrimental research practices;
- researchers, research sponsors, and research institutions should continue to develop and assess more effective education and other programs that support the integrity of research; further, they should leverage these partnerships to force the research through mutual learning and sharing of best practice.

Finally, those engaged in decision-making processes relating to environmental and occupational exposures should argue systematically for decisions that protect the most vulnerable and sensitive in society, such as children and pregnant women. Protecting the most vulnerable in society protects all and, besides, it is the responsibility of those in public health to advocate for those without a voice.

## What this commentary recognizes

Because, as David Michaels has noted, corporations provide “science for hire, period, and it is extremely lucrative” [29], in this commentary we bring attention to the undermining of occupational and environmental global public health through the insertion of “knowledge” through mechanisms of deliberate corporate manipulation that we label as malfeasance. Practices of corporate malfeasance include:

- Contamination of editorial boards of peer-reviewed scientific journals with industry apologists resulting in the publication of poorly-designed research studies that produce some biased results that mislead readers and flood the literature with invalid science;
- Interference with the activities of national regulatory bodies (e.g. USEPA, EFSA) and international review panels (e.g. WHO/IARC) and other independent organizations engaged in safeguarding occupational and public health;
- Constructing roadblocks, e.g. by capitalizing on uncertainty to undermine scientific consensus for much-needed government regulation of carcinogenic, endocrine-disrupting and/or immunotoxic agents widely present in the workplace and the environment, including air toxics, pesticides and toxic metals;
- The promotion of “causation” criteria that lack foundation and effectively block workers’ access to legal remedies for harms from occupational exposures resulting in morbidity and premature mortality.
- Violating standards of professional conduct by seducing reputable scientists with financial incentives that make them beholden to serve the corporate agenda.

This well-orchestrated assault on science must be met head-on and could be achieved by promoting and protecting the integrity of research. Further, avoiding influence by conflicted corporate affiliates in occupational and public health regulations would be needed. In so doing, the welfare of patients and society through quality medical and public health research and education would be more assured.

## Conclusions

The primary goals of medicine and public health activities are to:

- improve public health by fostering preventive strategies and providing beneficial care to patients and the public;
- conduct valid research;
- give advice to governmental decision-making bodies and in regulatory processes; and
- offer excellent medical and scientific education.

From the policy perspective, relevant scientific evidence comprises studies that are valid and sufficiently reliable. In pursuing these goals, individual professionals, health care institutions, and research organizations have obligations to put public health, workers and patient interests first, carry out unbiased research, critically appraise information, and serve as good role models of professional behavior for students.

Corporate interests have frequently influenced science by driving research agendas, manipulating the design, methods and conduct of research, and selectively publishing findings or affecting the interpretation of findings [7, 50]. Conflicting interests arise because, in many circumstances in modern medicine and public health, these goals and obligations are at risk of being compromised by the interest of financial gain.

Concern has also to be expressed owing to the fact that scientific integrity is frequently violated through research that is supported by individuals or corporations with conflicting interests, whose primary goal is often to protect markets for products or pollutants which have hazardous potential. The impact of corrupted science on subverting legislation, and in undermining policy-making, standard-setting and legal proceedings, is seen with greater frequency. This trend should alarm authorities, workers, consumers and the public at large.

Malfeasance has to be met by promoting and protecting the integrity of research, the welfare of patients and society, and the quality of medical and research education. Important measures are: open access to data, rigorous methodological standards, disclosure of conflicting interests and acknowledgement of bias in order to align with the principles of research integrity that are normative among researchers. Thus, academics, and public health researchers and practitioners should be alert to supporting industry initiatives disguised as ways to promote research integrity [9].

Effective strategies to avoid personal COIs are needed. These include the elimination of secondary interests, accompanied by prevailing full transparency, fairness, proportionality and accountability. Physicians and scientists involved in occupational and environmental health should never divert from the path of scientific integrity, assessments and communications. They should consistently strive for objectivity by impartially pursuing scientific truth with a view to public health protection.

## Abbreviations

COI: Conflict-of-interest
EFSA: European Food Safety Authority
EPA / USEPA: United States Environmental Protection Agency
FFP: Fabrication, falsification, or plagiarism
IARC: International Agency for Research on Cancer
SAP: Scientific Advisory Panel
WHO: World Health Organization
